# Evaluation of the difference between mean corpuscular haemoglobin concentration and mean cellular haemoglobin concentration in canine complete blood count assessed with an automated haematology analyser

**DOI:** 10.1111/jsap.70036

**Published:** 2025-10-29

**Authors:** M. G. Ferrari, S. Fasoli, K. Vasylyeva, E. Brini, F. Dondi, C. Agnoli

**Affiliations:** ^1^ Department of Veterinary Medical Sciences Alma Mater Studiorum – University of Bologna Bologna Italy

## Abstract

**Objectives:**

The study aimed (a) to establish the reference interval for Δmean corpuscular haemoglobin concentration‐mean cellular haemoglobin concentration in healthy dogs by ADVIA 2120 haematology analyser, (b) to identify the causes of increased Δmean corpuscular haemoglobin concentration‐mean cellular haemoglobin concentration in both healthy and diseased canine samples and (c) to establish a cut‐off value to prompt further diagnostic evaluations by practitioners.

**Materials and Methods:**

A retrospective study was conducted evaluating the medical records of dogs referred to a Veterinary University Hospital. Healthy dogs were prospectively included to establish reference interval for complete blood count variables comprising Δmean corpuscular haemoglobin concentration‐mean cellular haemoglobin concentration. Complete blood count reports of dogs who had both complete blood count and biochemistry performed concurrently were included. Complete blood counts were performed within 2 hours of sample collection, and biochemical analyses were carried out on serum samples within 12 hours. The macroscopic sample alterations were retrieved, including haemolysis, icterus, lipaemia and their severity.

**Results:**

Reference interval for Δmean corpuscular haemoglobin concentration‐mean cellular haemoglobin concentration was established and ranged from −1.70 to 2.20 g/dL (90% confidence interval of the upper limit 1.92 to 2.50). The Δmean corpuscular haemoglobin concentration‐mean cellular haemoglobin concentration was significantly increased in lipaemic and haemolytic samples and significantly correlated with their severity. The frequency of samples with increased Δmean corpuscular haemoglobin concentration‐mean cellular haemoglobin concentration (≥2.5 g/dL) was significantly higher in lipaemic and haemolytic samples and in patients undergoing corticosteroid therapy or affected by Cushing’s syndrome.

**Clinical Significance:**

An increase in Δmean corpuscular haemoglobin concentration‐mean cellular haemoglobin concentration in dogs is often associated with macroscopic sample alterations, mainly due to lipaemia, haemolysis, or both. When a Δmean corpuscular haemoglobin concentration‐mean cellular haemoglobin concentration value of >2.5 g/dL is noted, laboratories and practitioners should carefully evaluate the samples for any evidence of haemolysis or lipaemia.

## INTRODUCTION

Haemoglobin concentration (HGB) measurement includes different approaches: manual laboratory‐based methods (e.g., cyanmethaemoglobin method), automated haematology analysers, haemoglobinometers or quantitative colour scales (Karakochuk et al., 2019). Nowadays, haematology analysers are the main and most widely used tools to determine HGB using colourimetric methods (Karakochuk et al., 2019; Kunicka et al., 2001; Reichenwallner et al., 2021). Starting from the HGB value, the common haematology analysers provide the mean cellular haemoglobin concentration (MCHC) (in g/dL) which is calculated as follows: HGB/(RBC × MCV) × 1000, where RBC is the total number of red blood cells (×10^6^/μL) and MCV is the mean cellular volume (in fL) (Stockham & Scott, 2008a).

The ADVIA 2120 system combines two methods for assessing HGB: a colourimetric method (a modified cyanmethaemoglobin method) and a flow cytometry analysis, in which the haemoglobin content is measured based on a cell‐by‐cell analysis assessing the angular distribution of the light scattered by erythrocytes at a high angle (Tycko et al., 1985). This technology provides a novel variable, the mean cellular haemoglobin concentration (CHCM) (in g/dL) (Reichenwallner et al., 2021). When the difference between MCHC and CHCM (ΔMCHC‐CHCM) exceeds 1.9 g/dL, the ADVIA 2120 system generates an alarm flag based on data from human literature (Burgess et al., 1993; Diagnostics Siemens Healthcare, 2008). Starting from the value of CHCM, the analyser also provides the calculated haemoglobin value (cHGB) (in g/dL) by the following formula: (CHCM × RBC × MCV) ÷ 1000. The difference between HGB and cHGB is the ΔHGB which likely represents the free haemoglobin in the sample (Diagnostics Siemens Healthcare, 2008).

In routine analyses, we often encounter pre‐analytical sample alterations such as haemolysis (either in vitro or in vivo), lipaemia, or icterus. These alterations can lead to inaccuracies in colourimetric HGB measurement, resulting in a false increase in MCHC (Tvedten, 2022), unlike CHCM which is directly measured and largely unaffected by these interferences (Diagnostics Siemens Healthcare, 2008). Haemolysis occurs when erythrocytes are lysed in vivo or in vitro leading to the release of haemoglobin in the plasma. Lipaemia may result from elevated endogenous lipid concentration which occurs in conditions such as pancreatitis, cholestasis, diabetes mellitus, and nephrotic syndrome (Stockham & Scott, 2008b). Moreover, exogenous factors such as recent meal intake, or a high fat diet and administration of propofol could contribute to increased lipid concentration (Chagas et al., 2021; Stockham & Scott, 2008b); additionally, increased endogenous or exogenous corticosteroid levels can lead to hyperlipoproteinemia and the resulting lipaemia (Stockham & Scott, 2008b). Lastly, icterus refers to the yellow colour of the sample resulting from elevated total bilirubin concentration, mainly associated with liver disease or in vivo haemolysis (Gulati et al., 2022; Hosseini et al., 2014; Zandecki et al., 2007). Furthermore, aside from the plasma abnormalities mentioned earlier, it has been noted that plasma with high protein content (e.g., hyperglobulinemia) interferes with haemoglobin assessment, leading to an overestimation of HGB (Cornbleet, 1983; Gokcebay et al., 2011; Gulati et al., 2022).

To the best of the authors’ knowledge, no cut‐off value indicating a significant increase of ΔMCHC‐CHCM has been reported in veterinary medicine, nor has the impact of the aforementioned plasma alterations on this value been previously described. Therefore, this study primarily aimed to evaluate the main causes behind the increase of ΔMCHC‐CHCM in the complete blood count (CBC) obtained with the ADVIA 2120 haematology analyser in dogs admitted to a veterinary hospital. A secondary aim was to calculate the reference interval (RI) for ΔMCHC‐CHCM to suggest a cut‐off for this variable to alert practitioners when further diagnostic evaluations are required.

## MATERIALS AND METHODS

This was a retrospective study conducted using CBC results of dogs admitted to the Veterinary University Hospital (VUH) of the Department of Veterinary Medical Sciences, *Alma Mater Studiorum* – University of Bologna.

### Haemoglobin measurement

All the CBCs of this study were analysed with ADVIA 2120 (Siemens Healthcare Diagnostics, Tarrytown, New York). This haematology analyser assesses the HGB measurement by a colourimetric cyanmethemoglobin method and a flow cytometric method (Diagnostics Siemens Healthcare, 2008). The first method is based on the erythrocyte lysis to release haemoglobin: the iron contained in the haemoglobin is oxidised from the ferrous to the ferric state and then combined with cyanide in the HGB reagent to form the final reaction product; thus, the optical measurements are acquired colourimetrically using wavelengths of 565 or 546 nm (Diagnostics Siemens Healthcare, 2008; Malin et al., 1992; Reichenwallner et al., 2021). The second method involves a constant volume of cell suspension passing from the erythrocytes’ reaction chamber, named “RBC reaction chamber”, to the flow cell, where signals generated by each cell are measured using low‐angle scatter (2° to 3°) and high‐angle scatter (5° to 15°) (Diagnostics Siemens Healthcare, 2008). A pair of low‐angle (2° to 3°) and high‐angle (5° to 15°) scatter signals is used to analyse erythrocytes; then, the low‐angle scatter measurement is converted into cell volume, while the high‐angle scatter one is converted into haemoglobin concentration within the erythrocytes (Diagnostics Siemens Healthcare, 2008).

### Healthy dogs

Healthy blood donor dogs at our Transfusion medicine service, as well as healthy dogs owned by the VUH staff, were prospectively included in the study to establish RI for CBC variables including MCHC, CHCM and ΔMCHC‐CHCM. Dogs were defined as healthy based on their signalment, clinical history, physical examination, and clinicopathologic results. Clinicopathologic evaluation of these dogs included CBC (ADVIA 2120, Siemens Healthcare Diagnostics, Tarrytown, New York) with microscopic blood smear examination stained with May‐Grunwald Giemsa (Merck KGaA, Darmstadt, Germany). In addition to CBC, samples collected from these dogs underwent serum chemistry (AU 480, Olympus/Beckman Coulter, Brea, California) and urinalysis, including manual refractometer assessment of urine specific gravity, dipstick tests (Combur‐Test 10 UX, Roche, Switzerland) evaluated with an automated analyser (URISYS 1100, Roche, Switzerland), and microscopic examination of the urine sediment. All serum or plasma samples were required to exhibit no colour alterations such as haemolysis, icterus, or lipaemia. Dogs had to test negative for infectious diseases such as borreliosis, ehrlichiosis, anaplasmosis, and filariasis (SNAP 4DX antigen test, IDEXX Laboratories, Inc., Westbrook, Maine, USA), for *Leishmania* spp. and *Ehrlichia canis* by indirect fluorescent antibody test (MegaFLUO® LEISH, and MegaFluo® Ehrlichia canis, Diagnostik GmbH, Hörbranz, Austria). The inclusion and sampling of healthy dogs were approved by the local Scientific Ethical Committee (Protocol No. 57790, March 3, 2023).

### Data extraction from the hospital population

Medical records of dogs admitted to our VUH in a two‐year period (2020 to 2022) were retrospectively retrieved regardless of the number of CBCs performed per patient. Only dogs who had undergone blood sampling for both CBC (ADVIA 2120, Siemens Healthcare Diagnostics, Tarrytown, New York) with microscopic blood smear evaluation stained with May‐Grunwald Giemsa (Merck KgaA, Darmstadt, Germany) and serum chemistry analysis (AU 480; 220 Olympus/Beckman Coulter, Brea, CA, United States) from the same blood sampling were included. CBCs were performed within 2 hours of sample collection, and biochemical analyses were carried out on serum samples within 12 hours of collection and centrifugation. All cases wherein the ΔMCHC‐CHCM exceeded the 90% confidence interval (CI) of the upper limit (UL) of the RI (2.5 g/dL) (see the ‘Results’ section) were identified, and their medical records were retrieved. Administered therapies, with a particular focus on the use of steroids, were also reviewed. Selected subjects were classified into seven diagnostic groups according to their final diagnosis, encompassing the following disease categories: endocrine, gastrointestinal, haematologic, neurological, neoplastic, trauma and surgery, and miscellaneous (dermatological, genitourinary, respiratory, infectious, and idiopathic disease).

### Macroscopic samples characteristics

**FIG 1:**
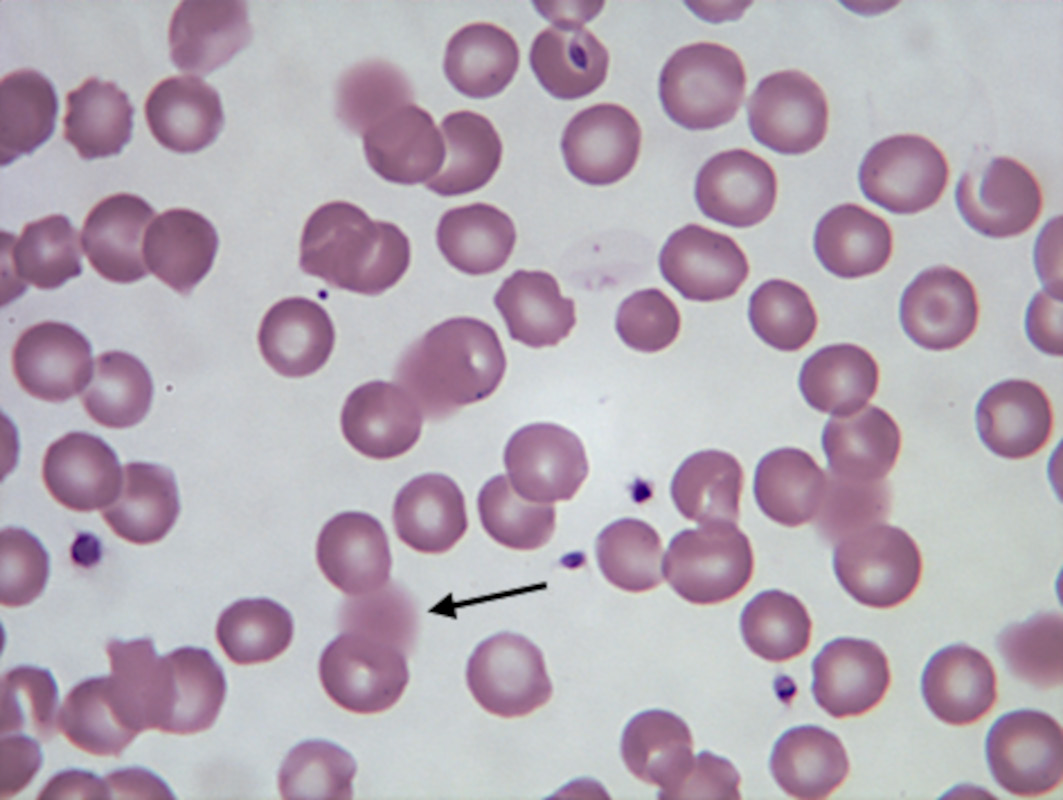
Erythrocyte lysis related to lipaemia (black arrow): numerous lysed red blood cells are observed due to heightened erythrocyte lysis induced by lipaemia during the blood film preparation. Stained with May–Grünwald Giemsa. Magnification: 1000×

**FIG 2:**
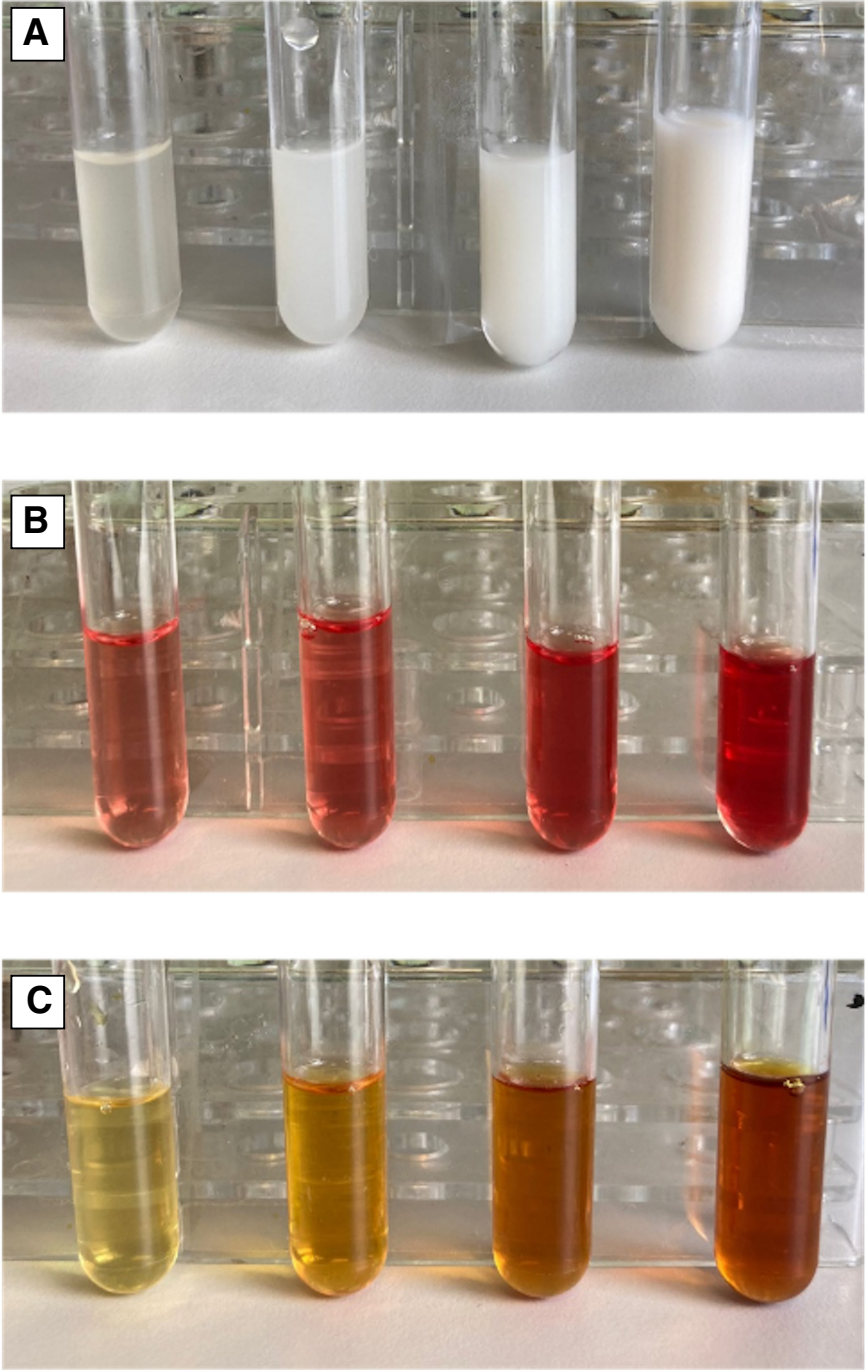
The internal laboratory grading system used to assess the presence of lipaemia (A), haemolysis (B), and icterus (C) from 1+ (left) to 4+ (right)

The presence of lipaemia, icterus, or haemolysis in the blood and serum samples of the dogs selected for the study was retrospectively analysed. Lipaemia was evaluated on both serum and blood samples and defined as follows: (a) the presence of a macroscopic lipaemic serum sample, (b) the presence of lipaemia‐related erythrocyte lysis observed during the microscopic evaluation of the blood smear (Harvey et al., 2012) (Fig 1), or both. A serum sample was considered icteric or haemolytic if the sample macroscopically exhibited these alterations. Macroscopic serum appearances, such as the presence of haemolysis, icterus, and lipaemia, were routinely recorded and graded by the operator from 1 to 4 (Fig 2), and results were grouped accordingly. Since colour alterations in the samples could be present simultaneously, each serum pigmentation was graded; for example, a lipaemic sample could exhibit a certain grade of haemolysis, and both were assessed accordingly. Over time, repeated samples obtained from the same dog were included if they met the inclusion criteria.

### Statistical analysis

Reference intervals for the haematological variables were determined and included in particular HGB, cHGB, MCV, RBC, mean corpuscular haemoglobin (MCH), MCHC, CHCM, ΔMCHC‐CHCM and ΔHGB. The selection of animals for the establishment of RI was performed following the American Society of Veterinary Clinical Pathology guidelines (Friedrichs et al., 2012). The reference intervals were calculated using a non‐parametric method (2.5th and 97.5th percentiles) (Friedrichs et al., 2012; Geffré et al., 2011). Data distribution was tested graphically and by using the Shapiro–Wilk normality test or the D’Agostino–Pearson test. Data were reported as mean ± standard deviation (SD) or median and range (minimum–maximum value), depending on normal or non‐normal distribution, respectively. Non‐parametric variables were compared using the Mann–Whitney *U* or Kruskal–Wallis test. Correlations were evaluated using Spearman’s rank correlation coefficient. Categorical variables were compared using the chi‐squared test or Fisher’s exact test. Results with a *P*‐value (*P*) of ≤.05 were considered statistically significant. Statistical evaluations were performed using commercially available software (MedCalc Software Ltd, Ostend, Belgium; https://www.medcalc.org; 2020; GraphPad Prism, version 8.4.2; GraphPad software, La Jolla, CA, USA).

## RESULTS

### Reference intervals

One hundred and twenty‐one dogs were enrolled in the healthy group to establish the RIs. The median age of these dogs was 3.9 years (range, 1 to 10.2 years), and the median weight was 25 kg (range, 1.3 to 78 kg). Forty‐seven dogs out of 121 were intact males (38.8%), 12/121 (9.9%) were neutered males, 28/121 (23.1%) were intact females, and 34/121 (28.1%) were spayed females. Thirty‐six dogs out of 121 (29.8%) were mixed breed, while 85/121 (70.2%) were purebred dogs (Table S1). The RI for ΔMCHC‐CHCM was −1.70 to 2.20 (90% CI of the UL 1.92 to 2.50), and the value of ≥2.5 g/dL was used as the threshold to select samples with increased ΔMCHC‐CHCM. Descriptive data and RIs of the variables cited in this study are reported in Table 1.

**Table 1:** Descriptive statistics and reference intervals for selected complete blood count variables in healthy dogs (*n* = 121) determined by ADVIA 2120 haematology analyser

Variable	Mean ± SD	Median (range)	RI LL (90% CI)	RI UL (90% CI)
CHCM (g/dL)	34.50 ± 0.75	34.45 (32.60 to 36.40)	32.90 (32.82 to 33.23)	36.00 (35.78 to 36.17)
cHGB (g/dL)	17.05 ± 1.68	16.80 (13.10 to 20.60)	14.00 (13.29 to 14.19)	20.21 (20.00 to 20.81)
HGB (g/dL)	17.13 ± 1.56	17.10 (13.60 to 20.70)	13.70 (13.64 to 14.39)	20.44 (19.76 to 20.60)
MCH (pg)	24.49 ± 1.08	24.50 (21.70 to 27.00)	22.40 (22.09 to 22.67)	26.70 (26.42 to 26.96)
MCHC (g/dL)	34.70 ± 1.20	34.85 (31.00 to 37.20)	32.29 (32.03 to 32.65)	36.90 (36.75 to 37.37)
RBC (×10^6^/μL)	7.02 ± 0.70	6.96 (5.53 to 8.83)	5.61 (5.43 to 5.79)	8.38 (8.18 to 8.56)
MCV (fL)	70.53 ± 3.2	70.50 (60.10 to 79.00)	63.60 (63.34 to 65.03)	76.90 (76.04 to 77.72)
ΔHGB	0.09 ± 0.47	0.10 (−1.30 to 1.10)	−0.8 (−0.95 to 0.67)	1 (0.89 to 1.15)
ΔMCHC‐CHCM	0.23 ± 0.97	0.30 (−2.30 to 2.50)	−1.70 (−1.92 to 1.38)	2.20 (1.95 to 2.50)

CHCM Cellular haemoglobin concentration mean, cHGB Calculated haemoglobin, CI Confidence interval, HGB Haemoglobin, LL Lower limit, MCH Mean corpuscular haemoglobin, MCHC Mean corpuscular haemoglobin concentration, MCV Mean corpuscular volume, RBC Red blood cell, RI Reference interval, SD Standard deviation, UL Upper limit, ΔHGB Difference between HGB and cHGB, ΔMCHC‐CHCM Difference between MCHC and CHCM

### Hospital population data

During the study period, 13,200 CBC from 4049 dogs were available for the study. According to the inclusion criteria, 2778 CBC results were excluded because of the absence of contextual serum chemistry analyses. Additionally, 382 CBCs were excluded due to incomplete data (e.g., lack of blood smear evaluation), resulting in a final sample size of 10,040 samples collected from 4049 dogs. The median age of these dogs was 8.4 years (0.1 to 20.8 years); 1417/4049 were intact males (35%), 582/4049 (14.4%) were neutered males, 762/4049 (18.8%) were intact females, and 1288/4049 (31.8%) were spayed females. Moreover, 1293/4049 (31.9%) were mixed breeds, while 2756/4049 (68.1%) were purebred dogs (Table S2). Based on the threshold for ΔMCHC‐CHCM here established, 142/10,040 (1.4%) samples from 113 dogs had a ΔMCHC‐CHCM ≥2.5 g/dL. From 16/113 dogs, a median of 2.5 samples (range 2 to 5) were collected on separate days.

### Effect of lipaemia, haemolysis and icterus on ΔMCHC‐CHCM in the overall population

A total of 899/10,040 samples (8.95%) were reported as exclusively lipaemic (i.e., serum features, presence at blood smear of signs indicating lipaemia‐related erythrocyte lysis, or both). Additionally, 620/10,040 (6.18%) samples exhibited only haemolysis, while 265/10,040 (2.64%) were exclusively icteric.

**FIG 3:**
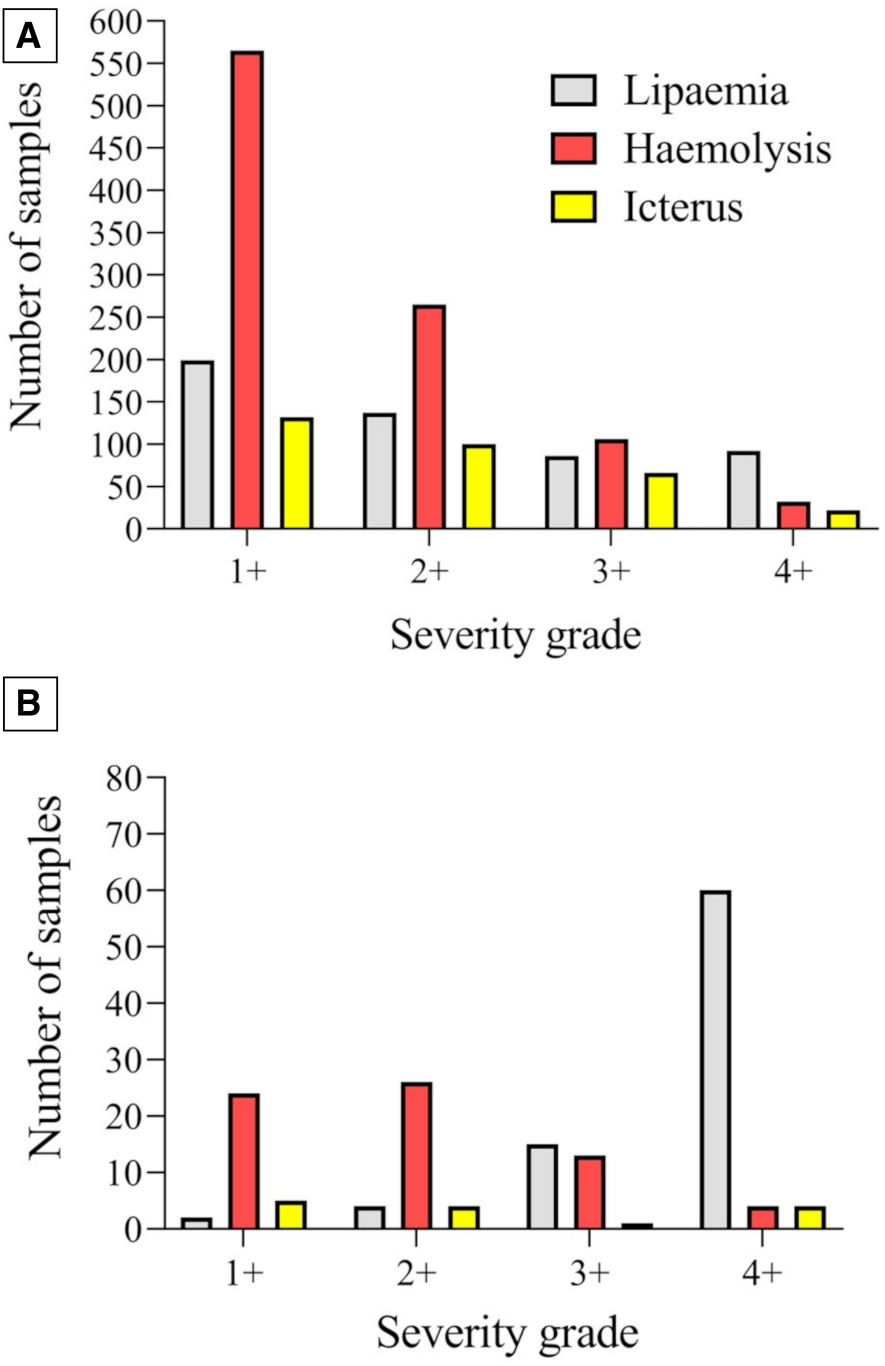
(A) Distributions of the severity grade of lipaemia, haemolysis, and icterus in the overall serum samples. (B) Distributions of the severity grade of lipaemia, haemolysis, and icterus in samples with ΔMCHC‐CHCM ≥2.5 g/dL

**FIG 4:**
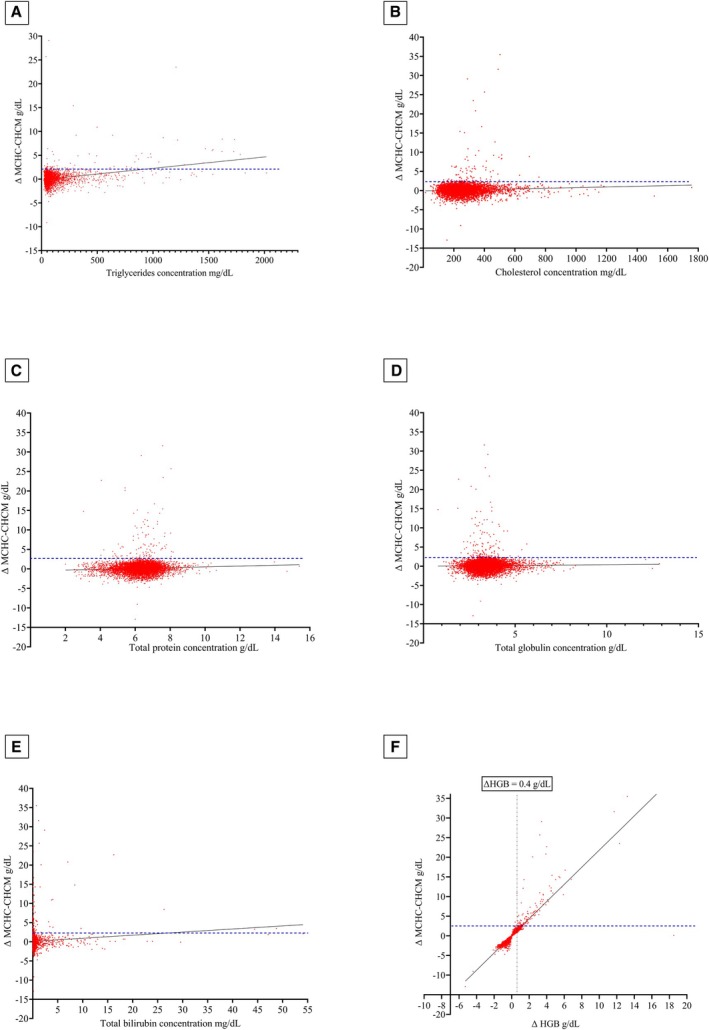
Correlation between ΔMCHC‐CHCM and (A) triglycerides (*r* = 0.10; *P* < .001), (B) total cholesterol (*r* = 0.02; *P* = .11), (C) total protein (*r* = 0.07; *P* < .001), (D) globulin (*r* = 0.04; *P* < .001), (E) total bilirubin (*r* = −0.004; *P* = .70) concentrations, (F) ΔHGB (*r* = 0.98; *P* < .001). The blue dashed line represents the value of ΔMCHC‐CHCM = 2.5 g/dL. The black continuous line represents the trend line

Several combinations of abnormalities were observed in some serum samples concurrently. Specifically, 317/10,040 (3.16%) samples were haemolytic and lipaemic, 30/10,040 (0.30%) samples were haemolytic and icteric, and 24/10,040 (0.24%) samples were lipaemic and icteric. Icterus, lipaemia, and haemolysis were simultaneously reported in only 1/10,040 (0.01%) sample, while no anomalies were reported in 7884 out of 10,040 (78.43%) serum samples. The distributions of lipaemia, haemolysis, and icterus grading are described in Fig 3A. The ΔMCHC‐CHCM was significantly increased in lipaemic samples compared to non‐lipaemic ones (median 0.5, range −3.2 to 35.5 g/dL; median 0.1, range −12.9 to 29.1 g/dL, respectively; *P* < .001), as well as in haemolytic samples compared to non‐haemolytic ones (median 0.3, range −4.7 to 25.7 g/dL; median 0.1, range −12.9 to 35.5 g/dL, respectively; *P* < .001). No difference between icteric and non‐icteric samples (median 0.1, range −3.6 to 29.10 g/dL; median 0.1, range −12.9 to 35.5 g/dL, respectively; *P* = .86) was detected (Table 2). The greater the increase in ΔMCHC‐CHCM, the more severe the lipemia and haemolysis (*r* = 0.17; *P* < .001 and *r* = 0.09; *P* < .001, respectively). A significant correlation was also found between ΔMCHC‐CHCM and triglyceride, globulin, and total protein concentrations. No correlation with ΔMCHC‐CHCM was detected for total cholesterol and total bilirubin concentration. A significant correlation was found between ΔHGB and ΔMCHC‐CHCM (Fig 4).

**Table 2:** ΔMCHC‐CHCM in lipaemic, haemolytic, and icteric samples and their difference in means and 95% confidence intervals

	Lipaemia				Haemolysis				Icterus			
	Lipaemic samples	Non‐lipaemic samples	Difference in means (95% CI)		Haemolytic samples	Non‐haemolytic samples	Difference in means (95% CI)		Icteric samples	Non‐icteric samples	Difference in means (95% CI)	
ΔMCHC‐CHCM (g/dL)	0.5 (3.2 to 35.5)	0.1 (−12.9 to 29.1)	−0.78 (−0.8640 to −0.6958)	***P* < .001**	0.3 (−4.7 to 25.7)	0.1 (−12.9 to 35)	0.09 (−0.0075 to 0.1815)	***P* < .001**	0.1 (−3.6 to 29.10)	0.1 (−12.9 to 35.5)	−0.30 (−0.4404 to −0.1669)	*P* = .86

Data are indicated as median and range (minimum–maxim value). Those in bold are statistically significant results

CHCM Cellular haemoglobin concentration mean, CI Confidence interval, MCHC Mean corpuscular haemoglobin concentration; ΔMCHC‐CHCM Difference between MCHC and CHCM

### Effect of lipaemia, haemolysis and icterus in samples with ΔMCHC‐CHCM ≥2.5 g/dL

Of the samples with ΔMCHC‐CHCM ≥2.5 g/dL, a total of 35/143 (24.47%) were reported as exclusively lipaemic, 8/143 (5.60%) samples exhibited only haemolysis, and 5/143 (3.50%) were exclusively icteric. Various combinations of abnormalities were reported to be concurrent: 55/143 (38.46%) samples were haemolytic and lipaemic, 5/143 (3.50%) were lipaemic and icteric, and 4/143 (3.49%) were haemolytic and icteric. No serum alterations were reported in 31/143 (21.70%) of these samples. The distributions of lipaemia, haemolysis, and icterus grade in samples with ΔMCHC‐CHCM ≥2.5 g/dL are described in Fig 3B.

**FIG 5:**
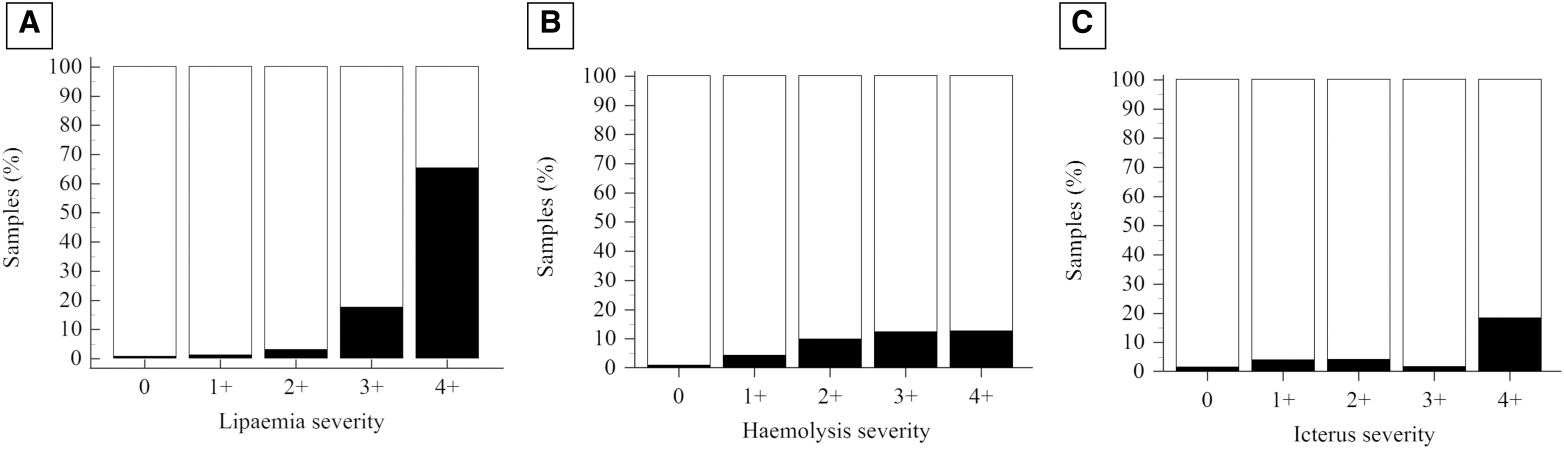
The frequency of samples with ΔMCHC‐CHCM ≥2.5 g/dL (black part of the column), evaluated for each grading of (A) lipaemia, (B) haemolysis, and (C) icterus. Note the increase in the black part of the column (samples with ΔMCHC‐CHCM ≥2.5 g/dL) when the severity of the grade increased

In lipaemic samples, the proportion of CBC reports with a ΔMCHC‐CHCM ≥2.5 g/dL was 66%; while in the haemolytic samples, the proportion of ΔMCHC‐CHCM ≥2.5 g/dL was 45%. These proportions were significantly higher when compared to non‐lipaemic (12%) and non‐haemolytic (9%) counterparts (*P* < .001) (Table 3). A statistically significant difference was also observed between icteric and non‐icteric samples (10% vs. 3%, *P* < .001) (Table 3). As the grade of lipaemia increased, the frequency of samples with ΔMCHC‐CHCM ≥2.5 g/dL also increased. Similarly, an increase in the grade of haemolysis and icterus also led to an increase in the frequency of samples with a ΔMCHC‐CHCM >2.5 g/dL (*P* < .001) (Fig 5).

**Table 3:** The frequency of samples with ΔMCHC‐CHCM ≥2.5 g/dL, their difference in means, and their 95% confidence intervals (CI) is reported

	Lipaemia				Haemolysis				Icterus			
	Lipaemic samples	Non‐lipaemic samples	Difference in means (95% CI)		Haemolytic samples	Non‐haemolytic samples	Difference in means (95% CI)		Icteric samples	Non‐icteric samples	Difference in means (95% CI)	
ΔMCHC‐CHCM ≥2.5 g/dL	66%	12%	54% (38.3% to 64.9%)	***P* < .001**	45%	9%	36% (21.8% to 48.7%)	***P* < .001**	10%	3%	7% (−2% to 32%)	***P* < .001**

Those in bold are statistically significant results

CHCM Cellular haemoglobin concentration mean, CI Confidence interval, MCHC Mean corpuscular haemoglobin concentration, ΔMCHC–CHCM, difference between MCHC and CHCM

### Medical records of dogs with ΔMCHC‐CHCM ≥2.5 g/dL

Dogs with ΔMCHC‐CHCM above 2.5 g/dL were distributed in the following disease categories: neoplastic 23/113 (20.4%), endocrine 21/113 (18.6%) (10/21 were affected by Cushing’s syndrome), trauma and surgery 12/113 (10.6%), gastrointestinal 12/113 (10.6%), neurologic 12/113 (10.6%), haematologic 12/113 (10.6%), and miscellaneous 21/113 (18.6%). Out of 113 dogs, 31 (27.4%) were receiving corticosteroid therapy, and 10 (8.8%) were diagnosed with Cushing’s syndrome. Therefore, a total of 41 out of 113 dogs (36.2%) were either undergoing corticosteroid treatment or affected by Cushing’s syndrome. Results of ΔMCHC‐CHCM were not different among disease categories (*P* = .12), while the ΔMCHC‐CHCM was significantly increased in samples obtained from dogs treated with corticosteroids or affected by Cushing’s syndrome (median 5.2; range 2.5 to 29.1 g/dL) compared to the others (median 3.4; range 2.5 to 15.4 g/dL; difference in means: −2168; 95% CI: −37.555 to −0.5802; *P* = .007). No difference in the ΔMCHC‐CHCM value between dogs treated with corticosteroids and dogs affected by Cushing’s syndrome (median 5.2, range 2.5 to 29.1 g/dL; median 5.5, range 2.5 to 12.1 g/dL, respectively; difference in means and 95% CI, −0.7426, −4.7546 to 3.2694; *P* = .67) was detected.

## DISCUSSION

The aim of this study was to identify the possible causes contributing to an increase in ΔMCHC‐CHCM in canine blood samples from VUH patients and analysed with a flow‐cytometry‐based automated haematology analyser. To achieve this goal, the ΔMCHC‐CHCM RI was established as −1.7 to 2.2 g/dL; secondly, a retrospective analysis of CBC results processed with the automated analyser in use at our VUH (ADVIA 2120, Siemens Healthcare Diagnostics, Tarrytown, New York) has been conducted to investigate the potential causes affecting this variable. A significant difference between MCHC and CHCM is reported for humans only when it exceeds 1.9 g/dL, and this cut‐off value is used as a helpful tool to guide the decision‐making and interpretation of laboratory results (Burgess et al., 1993). Conversely, no similar data are reported in veterinary medicine for ΔMCHC‐CHCM to alert practitioners when detailed evaluations are required for patients. In order to investigate this variable in the dog and to improve its specificity, the authors decided to consider as the ΔMCHC‐CHCM cut‐off value the UL of 90% CI of ΔMCHC‐CHCM UL of the RI, that is 2.5 g/dL.

Our results show that ΔMCHC‐CHCM was significantly increased in the presence of a macroscopic lipaemic sample. Although a significant correlation between triglyceride concentration and ΔMCHC‐CHCM was found, no correlation was pointed out with cholesterol concentration. Lipaemia or hyperlipidaemia is characterised by an elevated concentration of lipoproteins, mainly very low‐density lipoproteins (VLDLs) and chylomicrons (Stockham & Scott, 2008b). These particles are rich in triglycerides or cholesterol (Remaley et al., 2014; Stockham & Scott, 2008b). A triglyceride concentration >300 mg/dL in centrifuged samples can result in a cloudy appearance, which turns into opacity that is easily seen by the naked eye when the concentration is >600 mg/dL (Remaley et al., 2014). This turbidity, caused by lipaemia, induces the dispersion of light throughout the entire visual spectrum (300 to 700 nm), with a proportional rise in intensity as the wavelength diminishes (Fernández Prendes et al., 2023). This condition can result in altered haemoglobin readings, thereby impacting the accuracy of the spectrophotometric method (Gagné et al., 1977; Guder et al., 2000; Gulati et al., 2022). Therefore, it is conceivable that our lipaemic samples graded 3+ or 4+ might depict an increased concentration of chylomicrons and very low‐density proteins, which could cause interference in haemoglobin measurement due to sample turbidity and raise the MCHC. Moreover, it has been reported that haematology analysers using the photometry method for HGB measurement could give a reliable HGB result up to 264 mg/dL of triglyceride concentration (Zandecki et al., 2007). Our graded 3+ or 4+ lipaemic samples had a median triglyceride concentration of 354 mg/dL (range 24 to 939), additionally supporting the inability of the haematology analyser to perform a reliable measurement of HGB in these samples. The absence of a correlation between cholesterol concentration and ΔMCHC‐CHCM might be justified since hypercholesterolemia does not cause increased serum turbidity.

In macroscopic haemolytic samples, ΔMCHC‐CHCM was higher than in non‐haemolytic ones, likely due to free haemoglobin resulting from haemolysis within the bloodstream (in vivo) or during and after the collection of blood (in vitro). According to previous reports, free haemoglobin concentrations up to 0.4 g/dL are not expected to affect haemoglobin measurements obtained using haematology analysers (Zandecki et al., 2007). All samples with ΔMCHC‐CHCM above 2.5 g/dL exhibited a corresponding increase in ΔHGB exceeding 0.4 g/dL. This finding suggests the possibility of free haemoglobin in these samples and justifies its misinterpretation as part of the total red blood cell haemoglobin, resulting in an erroneously elevated MCHC and consequently increased ΔMCHC‐CHCM.

Significant differences in ΔMCHC‐CHCM values between icteric and non‐icteric samples were not observed, and no correlation with total bilirubin concentration was evident. Typically, visible icterus is observed when the total bilirubin concentration exceeds 1.5 mg/dL (Stockham & Scott, 2008c). A previous study reported that concentrations of bilirubin between 25 and 35 mg/dL can cause interferences in HGB readings (Gulati et al., 2022). This might justify our result since only 3 out of 103 samples with ΔMCHC‐CHCM ≥2.5 g/dL had a total bilirubin concentration >25 mg/dL, two of which were >35 mg/dL (49 and 44 mg/dL). Nevertheless, the frequency of samples with ΔMCHC‐CHCM ≥2.5 significantly increased in icteric compared to non‐icteric samples; however, other concomitant serum alterations (e.g., lipaemia or haemolysis) could have influenced this data. We also observed a weak positive correlation between ΔMCHC‐CHCM and total protein and globulin concentrations; however, the low correlation coefficients suggest that these variables are unlikely to have a meaningful impact; therefore, the biological relevance of this association remains questionable. It has been reported that an increase in sample turbidity, that is, due to the increased concentration of protein in the plasma, might lead to spurious increases in HGB (Cornbleet, 1983; Gokcebay et al., 2011). Human patients with elevated concentrations of monoclonal immunoglobulins (Ig) such as IgM, IgA, or IgG, as seen in conditions like multiple myeloma, might experience a false increase in haemoglobin measurements when samples are analysed using automated techniques. This is due to the interaction between these elevated Ig concentrations and the reagents in the lysis solution, resulting in the formation of an optically dense precipitate (Goodrick et al., 1993; McMullin et al., 1995; Roberts et al., 2000). It is possible that unexpected interactions occurred between our samples, characterised by high total protein concentration, and the haematology analyser’s reagents, potentially affecting haemoglobin readings. However, the possibility that other factors could have influenced this outcome should be pointed out, and further studies are needed to clarify these findings.

**FIG 6:**
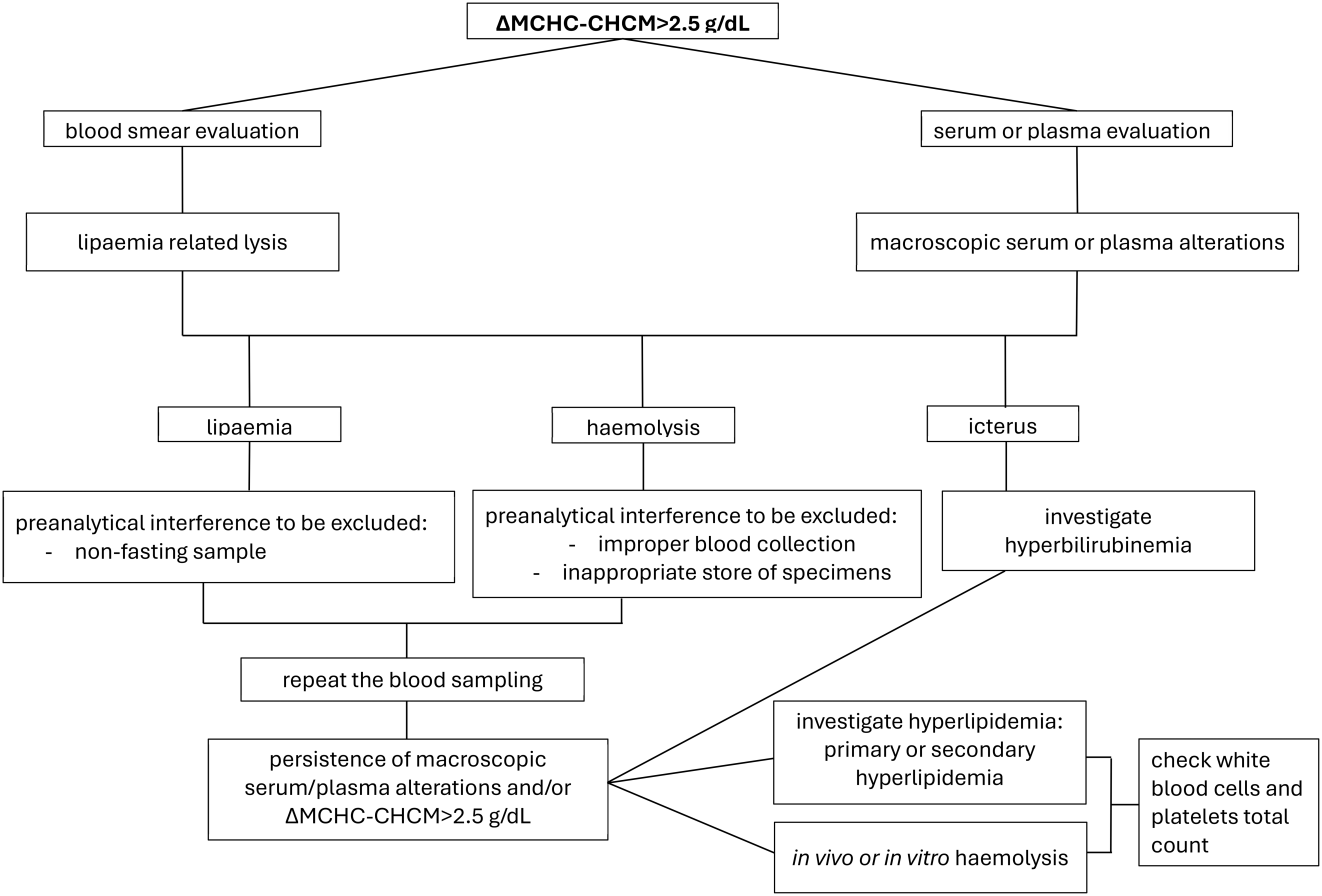
Decision‐making summary algorithm when samples with ΔMCHC‐CHCM ≥2.5 g/dL are encountered in dogs

Our study showed that the frequency of ΔMCHC‐CHCM above 2.5 g/dL was significantly increased in dogs undergoing corticosteroid therapy, affected by Cushing’s syndrome, or both. Corticosteroids, whether endogenous or exogenous, can induce hyperlipoproteinemia by stimulating lipoprotein production, promoting the release of fatty acids from hepatocytes, inducing insulin resistance, and subsequently diminishing lipoprotein activity (Stockham & Scott, 2008b). The liver converts these free fatty acids into triglycerides, packaging them into very low‐density lipoprotein (VLDL) particles (Behrend, 2014). In these conditions, dogs likely experienced an elevation in VLDL that prompted lipaemic samples with increased turbidity to interfere with the haemoglobin reading. Considering our findings, we propose a summary algorithm to assist the practitioner in decision‐making needed when a sample with ΔMCHC‐CHCM ≥2.5 g/dL is encountered (Fig 6).

This study had several limitations that should be taken into account when interpreting its results. Primarily, we selected the upper limit of the 90% confidence interval of the UL of the ΔMCHC‐CHCM RI, set at 2.5 g/dL, as the cut‐off value. While this choice could have led to the exclusion of certain cases, it was made to minimise the occurrence of false positives potentially associated with further unnecessary investigations (Cracknell et al., 2022). A second limitation of this study is related to its retrospective nature, limiting the possibility of verifying the accuracy of the reported sample alterations and potentially explaining why certain samples exhibited a ΔMCHC‐CHCM ≥2.5 g/dL without observable colour alterations. Nevertheless, the high number of samples included in the study may have helped to overcome this limitation. Additionally, since the reporting and grading of alterations are highly operator‐dependent, evaluating these changes using automated analysis to identify haemolysis, icterus, and lipaemia indices would be preferable, although even this method is not totally accurate and is susceptible to many sample interferences (Nikolac Gabaj et al., 2018). Moreover, not all serum samples were analysed for triglycerides, cholesterol, total proteins, and bilirubin concentrations, which might have influenced the results, and a prospective study would be necessary to assess the impact of those analytes of difficult explanation, such as total proteins. Finally, the presence of concomitant colour abnormalities could have had an impact on our results, making it difficult to identify the exact role of each specific colour alteration in some samples. For example, lipaemia is frequently associated with haemolysis due to changes in RBC membranes caused by lipids (Dimeski et al., 2005).

In conclusion, an increase in ΔMCHC‐CHCM obtained with ADVIA 2120 in dogs is associated with relevant plasma colour alterations, mainly due to macroscopic lipaemia and haemolysis, and the frequency of this finding increases with the severity of the colour abnormalities. When clinicians or clinical pathologists encounter a ΔMCHC‐CHCM ≥2.5 g/dL, they should suspect haemolysis or lipaemia, confirm the occurrence by visual inspection of serum, plasma, or both, and investigate the possible underlying causes. Additionally, they should carefully evaluate other variables of the CBC, such as leucocyte and platelet counts, which can also be falsely elevated (Stockham & Scott, 2008b; Zandecki et al., 2007). Notably, ΔMCHC‐CHCM ≥2.5 g/dL should be considered a warning signal for macroscopical evaluation of the dog’s serum. It prompts careful consideration to avoid diagnostic investigations that might yield inaccurate results due to the impact of these alterations on method analysis.

### Author contributions

**M. G. Ferrari:** Conceptualization (equal); data curation (equal); writing – original draft (equal); writing – review and editing (equal). **S. Fasoli:** Conceptualization (equal); formal analysis (equal); writing – original draft (equal); writing – review and editing (equal). **K. Vasylyeva:** Conceptualization (equal); data curation (equal); writing – review and editing (equal). **E. Brini:** Investigation (equal); writing – review and editing (equal). **F. Dondi:** Project administration (equal); writing – review and editing (equal). **C. Agnoli:** Conceptualization (equal); project administration (equal); writing – review and editing (equal).

### Conflict of interest

The authors declared no potential conflicts of interest with respect to the research, authorship, and/or publication of this article.

## Supporting information


Table S1.

Table S2.

## Data Availability

The data that support the findings of our study are available from the corresponding author upon reasonable request.
