# Supplementary material for: Evaluation of the difference between mean corpuscular haemoglobin concentration and mean cellular haemoglobin concentration in canine complete blood count assessed with an automated haematology analyser
Source: J Small Anim Pract. 2025 Oct 29;67(3):243–53. doi: 10.1111/jsap.70036 (PMC12968496; doi:10.1111/jsap.70036)
Supplement: Supplementary file 1 — Table S1. [file JSAP-67-243-s002.docx]

| **Breed** | **Number of dogs** | **Percentage (%)** |
| --- | --- | --- |
| American Staffordshire Terrier | 3 | 2.47% |
| Australian Shepherd | 2 | 1.65% |
| Bernese mountain dog | 1 | 0.83% |
| French Bulldog | 12 | 9.91% |
| Boxer | 5 | 4.12% |
| Cavalier King Charles Spaniel | 1 | 0.83% |
| Chihuahua | 1 | 0.83% |
| Collie | 1 | 0.83% |
| Dobermann | 1 | 0.83% |
| English Bulldog | 17 | 14.05% |
| English Setter | 1 | 0.83% |
| Flat-coated Retriever | 2 | 1.65% |
| German Shepherd | 5 | 4.12% |
| Golden Retriever | 11 | 9.09% |
| Great dane | 2 | 1.65% |
| Kurzhaar | 1 | 0.83% |
| Labrador retriever | 6 | 4.96% |
| Lagotto romagnolo | 1 | 0.83% |
| Leonberger | 2 | 1.65% |
| Maremmano-Abruzzese Sheepdog | 1 | 0.83% |
| Mixed-breed | 36 | 29.75% |
| Pointer | 3 | 2.48% |
| Pug | 1 | 0.83% |
| Rough collie | 1 | 0.83% |
| Shih-tzu | 2 | 1.65% |
| St. Bernard dog | 1 | 0.83% |
| White Swiss Shepherd Dog | 1 | 0.83% |
| **Total** | **121** | **100%** |

Supplementary Table 1. Distribution of breeds in healthy dogs selected for reference intervals.
