# Supplementary material for: Evaluation of the difference between mean corpuscular haemoglobin concentration and mean cellular haemoglobin concentration in canine complete blood count assessed with an automated haematology analyser
Source: J Small Anim Pract. 2025 Oct 29;67(3):243–53. doi: 10.1111/jsap.70036 (PMC12968496; doi:10.1111/jsap.70036)
Supplement: Supplementary file 2 — Table S2. [file JSAP-67-243-s001.docx]

Supplementary Table 2. Distribution of breeds in the overall study group.

| **Breed** | **Number of dogs** | **Percentage (%)** |
| --- | --- | --- |
| Alaskan Malamute | 10 | 0.2% |
| Alpine Dachsbracke | 1 | 0.02% |
| American Bulldog | 1 | 0.02% |
| American Bully | 7 | 0.2% |
| American Staffordshire Terrier | 102 | 2.5% |
| Appenzeller Sennenhund | 2 | 0.05% |
| Australian Shepherd | 39 | 1,00% |
| Australian Silky Terrier | 1 | 0.02% |
| Basenji | 2 | 0.05% |
| Basset Hound | 11 | 0.3% |
| Bavarian Mountain Hound | 1 | 0.02% |
| Beagle | 31 | 0.8% |
| Beauceron | 1 | 0.02% |
| Belgian Shepherd Dog (Groenendael) | 11 | 0.3% |
| Bernese Mountain Dog | 42 | 1,00% |
| Bichon Frisé | 3 | 0.07% |
| Bloodhound | 1 | 0.02% |
| Bolognese | 27 | 0.7% |
| Border Collie | 63 | 1.6% |
| Border Terrier | 1 | 0.02% |
| Borzoi | 1 | 0.02% |
| Boston Terrier | 7 | 0.2% |
| French Bulldog | 120 | 3,00% |
| Boxer | 69 | 1.7% |
| Bracco Italiano | 9 | 0.2% |
| Briard | 2 | 0.05% |
| Brittany | 21 | 0.52% |
| Bull Terrier | 11 | 0.26% |
| Cairn Terrier | 2 | 0.05% |
| Cane Corso | 40 | 1,00% |
| Cao De Agua Portugues | 3 | 0.07% |
| Caucasian Shepherd Dog | 6 | 0.1% |
| Cavalier King Charles Spaniel | 53 | 1.3% |
| Central Asian Shepherd Dog | 2 | 0.05% |
| Chihuahua | 62 | 1.5% |
| Chow Chow | 5 | 0.1% |
| Cirneco Dell'etna | 5 | 0.1% |
| Cocker Spaniel | 51 | 1.3% |
| Collie | 6 | 0.1% |
| Czechoslvakian Wolfdog | 16 | 0.4% |
| Dachshund Kaninchen | 29 | 0.7% |
| Dalmatian | 3 | 0.07% |
| Deutscher Jagdterrier | 2 | 0.05% |
| Dobermann | 31 | 0.8% |
| Dogo Argentino | 9 | 0.2% |
| Dogue De Bordeaux | 7 | 0.2% |
| Drahthar | 3 | 0.07% |
| English Bulldog | 60 | 1.5% |
| English Setter | 73 | 1.8% |
| English Springer Spaniel | 12 | 0.3% |
| Finnish Lapphund | 1 | 0.02% |
| Finnish Spitz | 2 | 0.05% |
| Flat-Coated Retriever | 6 | 0.1% |
| Fox Terrier | 8 | 0.2% |
| Galgo Espanol | 20 | 0.5% |
| German Pinscher | 63 | 1.6% |
| German Shepherd | 145 | 3.6% |
| German Shorthaired Pointer | 18 | 0.4% |
| German Spitz | 5 | 0.1% |
| Giant Schnauzer | 5 | 0.1% |
| Golden Retriever | 146 | 3.6% |
| Gordon Setter | 1 | 0.02% |
| Great Dane | 12 | 0.3% |
| Great Swiss Mountain Dog | 1 | 0.02% |
| Greenland Dog | 1 | 0.02% |
| Greyhound | 8 | 0.2% |
| Griffon Bruxellois | 1 | 0.02% |
| Griffon Bleu De Gascogne | 4 | 0.1% |
| Hovawart | 5 | 0.1% |
| Hungarian Puli | 1 | 0.02% |
| Hungarian Pumi | 1 | 0.02% |
| Hungarian Wirehaired Vizsla | 13 | 0.3% |
| Ibizan Hound | 4 | 0.1% |
| Irish Setter | 11 | 0.3% |
| Irish Terrier | 1 | 0.02% |
| Irish Wolfhound | 1 | 0.02% |
| Italian Greyhound | 2 | 0.05% |
| Jack Russel Terrier | 98 | 2.4% |
| Japanese Akita Inu | 20 | 0.5% |
| Japanese Spitz | 2 | 0.05% |
| Kangal Çoban Köpeğ | 1 | 0.02% |
| Keeshond | 1 | 0.02% |
| Kurzhaar | 6 | 0.1% |
| Labrador Retriever | 219 | 5.4% |
| Lagotto Romagnolo | 46 | 1.1% |
| Leonberger | 7 | 0.2% |
| Lhasa Apso | 1 | 0.02% |
| Maltese | 107 | 2.6% |
| Manchester Terrier | 2 | 0.05% |
| Maremmano Abruzzese Sheepdog | 29 | 0.7% |
| Mastiff | 6 | 0.1% |
| Medium Poodle | 53 | 1.3% |
| Miniature Poodle | 25 | 0.6% |
| Miniature Schnauzer | 13 | 0.3% |
| Mixed-Breed Dog | 1294 | 32,00% |
| Newfoundland | 13 | 0.3% |
| Norwich Terrier | 1 | 0.02% |
| Nova Scotia Duck Tolling Retriever | 1 | 0.02% |
| Papillon | 5 | 0.1% |
| Pastore Della Lessinia-Lagorai | 1 | 0.02% |
| Pastore Della Sila | 1 | 0.02% |
| Pekingese | 6 | 0.1% |
| Pembroke Welsh Corgi | 2 | 0.05% |
| Pointer | 17 | 0.4% |
| Pomeranian Dog | 23 | 0.6% |
| Portuguese Podengo | 1 | 0.02% |
| Pug | 36 | 0.9% |
| Pyreanean Mountain Dog | 7 | 0.17% |
| Pyrenean Mastiff | 1 | 0.02% |
| Rhodesian Ridgeback | 5 | 0.1% |
| Rottweiler | 36 | 0.9% |
| Rough Collie | 2 | 0.05% |
| Saarlos Wolfdog | 3 | 0.07% |
| Saluki | 4 | 0.1% |
| Samoyed Dog | 6 | 0.1% |
| Scottish Terrier | 3 | 0.07% |
| Segugio Italiano | 20 | 0.5% |
| Segugio Maremmano | 5 | 0.1% |
| Shar-Pei | 7 | 0.2% |
| Shetland Sheepdog | 4 | 0.1% |
| Shiba Inu | 13 | 0.3% |
| Shih-Tzu | 36 | 0.9% |
| Siberian Husky | 13 | 0.3% |
| Spaniel (American Cocker) | 1 | 0.02% |
| Spanish Mastiff | 1 | 0.02% |
| Spinone Italiano | 3 | 0.07% |
| St. Bernard Dog | 6 | 0.1% |
| Standard Dachshund | 75 | 1.9% |
| Standard Poodle | 15 | 0.4% |
| Standard Schnauzer | 5 | 0.1% |
| Tibetan Mastiff | 1 | 0.02% |
| Tibetan Spaniel | 1 | 0.02% |
| Tibetan Terrier | 2 | 0.05% |
| Toy Poodle | 13 | 0.3% |
| Volpino Italiano | 15 | 0.4% |
| Weimaraner | 36 | 0.9% |
| Welsh Corgi (Cardigan) | 3 | 0.07% |
| Welsh Terrier | 1 | 0.02% |
| West Highland White Terrier | 26 | 0.6% |
| Whippet | 10 | 0.2% |
| White Swiss Shepherd Dog | 5 | 0.1% |
| Yorkshire Terrier | 65 | 1.6% |
| **Total** | **4049** | **100%** |
